# The role of biotechnology in the transition from plastics to bioplastics: an opportunity to reconnect global growth with sustainability

**DOI:** 10.1002/2211-5463.13119

**Published:** 2021-04-01

**Authors:** Micaela Degli Esposti, Davide Morselli, Fabio Fava, Lorenzo Bertin, Fabrizio Cavani, Davide Viaggi, Paola Fabbri

**Affiliations:** ^1^ Department of Civil, Chemical Environmental and Materials Engineering Alma Mater Studiorum Università di Bologna Italy; ^2^ Bologna Unit National Interuniversity Consortium for Materials Science and Technology (INSTM) Firenze Italy; ^3^ Department of Industrial Chemistry ‘Toso Montanari’ Alma Mater Studiorum Università di Bologna Italy; ^4^ Department of Agricultural and Food Sciences Alma Mater Studiorum Università di Bologna Italy

**Keywords:** bio‐based products, bioplastics, biopolymers, circular economy, renewable resources, sustainability

## Abstract

Building new value chains, through the valorization of biomass components for the development of innovative bio‐based products (BBPs) aimed at specific market sectors, will accelerate the transition from traditional production technologies to the concept of biorefineries. Recent studies aimed at mapping the most relevant innovations undergoing in the field of BBPs (Fabbri et al. 2019, Final Report of the Task 3 BIOSPRI Tender Study on Support to R&I Policy in the Area of Bio‐based Products and Services, delivered to the European Commission (DG RTD)), clearly showed the dominant position played by the plastics sector, in which new materials and innovative technical solutions based on renewable resources, concretely contribute to the achievement of relevant global sustainability goals. New sustainable solutions for the plastic sector, either bio‐based or bio‐based and biodegradable, have been intensely investigated in recent years. The global bioplastics and biopolymers market size is expected to grow from USD 10.5 billion in 2020 to USD 27.9 billion by 2025 (Markets and Markets, 2020, Bioplastics & Biopolymers Market by Type (Non‐Biodegradable/Bio‐Based, Biodegradable), End‐Use Industry (Packaging, Consumer Goods, Automotive & Transportation, Textiles, Agriculture & Horticulture), Region ‐ Global Forecast to 2025), and this high growth is driven primarily by the growth of the global packaging end‐use industry. Such relevant opportunities are the outcomes of intensive scientific and technological research devoted to the development of new materials with selected technical features, which can represent feasible substitutes for the fossil‐based plastic materials currently used in the packaging sectors and other main fields. This article offers a map of the latest developments connected to the plastic sector, achieved through the application of biotechnological routes for the preparation of completely new polymeric structures, or drop‐in substitutes derived from renewable resources, and it describes the specific role played by biotechnology in promoting and making this transition faster.

## Graphical abstract

Biotechnology holds the power to boost the transition from fossil‐based to bio‐based plastics by offering new sustainable routes for polymer synthesis based on waste biomasses.

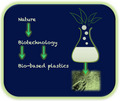

**Abbreviations**
BBPs: bio‐based products
CE: circular economy
LVHV: low‐volume high‐value

## Introduction

Over the last 150 years, the industrial system has been uniquely driven by a linear model, which is based on the production of goods starting from fossil raw materials, their commercialization, their use, and final disposal as waste to be discharged or incinerated [1, 2]. The traditional plastic sector shows an iconic representation of this linear model mainly in reference to the packaging field, with its large volume production almost exclusively intended for products for single or very short‐term use, followed by a fast transition to the waste state. For many reasons (including technical restrictions for commingled plastics recycling [3] or lack of infrastructure dedicated to recycling distributed over the territory), for a long time plastic waste was not considered a resource to be valued, but mainly a global problem giving rise to huge negative externalities. In 2014, *Valuing Plastic*, a report by the UN Environment Programme and the Plastics Disclosure Project, estimated the total natural capital cost of plastics in the consumer goods industry at USD 75 billion, of which USD 40 billion was related to plastic packaging, exceeding the profit pool of the plastic packaging industry [4]. In the face of such alarming indicators, the need for a fast transition for the plastic sector, shifting toward a sustainable model, is clearly understandable. The main routes toward a new circular economy (CE) for plastics have been already identified, and the Ellen MacArthur Foundation played an eminent role by giving their clear definition, reported in the document *The New Plastic Economy* published in 2016 [5]. Three main areas of intervention were identified: (a) creating an effective after‐use plastic economy, by promoting efficient recycling and a new design strategy inspired and driven by reuse and recyclability; (b) reducing the uncontrolled release of plastics into natural systems, by promoting more efficient waste collection and the development of infrastructure dedicated to waste treatment and valorization; (c) decoupling plastics from fossil resources, by promoting plastics derived from renewable resources.

Renewably sourced plastics decouple the production of plastics from fossil resources by sourcing the virgin feedstock either from captured greenhouse gases (GHG‐based) or biomass (bio‐based). Bio‐based plastics can be produced from different generations of feedstock [6, 7]:

1st generation: Biomass from plants that are rich in carbohydrates and that can be used as food or animal feed (e.g., sugar cane, corn, and wheat).
2nd generation: Biomass from plants that are not suitable for food or animal feed production. They can be either nonfood crops (e.g., cellulose) or waste materials from first‐generation feedstock (e.g., waste vegetable oil, bagasse, or corn stover).
3rd generation: Biomass derived from algae, which has a higher growth yield than either first‐ or second‐generation feedstock, and therefore has been allocated its own category.

Based on their physical and chemical properties, renewably sourced plastics can be divided into two categories: drop‐ins and completely new materials. Drop‐ins are identical replicates of the currently used traditional plastics, except that they are derived from renewable resources instead of being produced from fossil oil. The most relevant example is that of bio‐polyethylene (bio‐PE), which offers the same properties and features of standard PE, but is polymerized from ethylene monomer obtained by the dehydration of bioethanol, fermented from sugarcanes [8]. Drop‐ins can be directly introduced into the existing value chains, as they can deliver exactly the same level of technical performance as standard plastics. On the other hand, completely new bio‐based plastics can be derived from renewable resources without having a fossil‐based counterpart. This includes the examples of polylactic acid (PLA) or poly(hydroxyalkanoate)s (PHA), which offer a completely new set of properties but need a tailored approach for their introduction into production chains.

Bio‐based plastics indeed offer multiple advantages, ranging from decoupling plastics production from fossil resources, to decreasing carbon dioxide emissions. Bio‐based plastics could also act as a carbon sink throughout their life cycle. For plastics synthesized using carbon from captured GHG, this looks rather obvious [9]. For bio‐based plastics coming from vegetable source, this happens indirectly: Plants capture carbon dioxide from the atmosphere as they grow and this carbon is then harnessed in the polymer [10]. Bio‐PE GHG savings is up to 0.60 kg CO_2_e per kg for corn‐derived PE, and 3.4 kg CO_2_e per kg for switchgrass‐derived polyethylene compared to GHG emissions of standard fossil‐based PE [11].

In recent years, two main studies have been funded by the European Commission for the identification of innovative pathways from renewable feedstocks to innovative bio‐based products (BBPs), including biofuels and biochemicals, with biopolymers being a subcategory of the latter. The first study focused on the sugar platform and was completed in 2015 [12]. Its main objective was to provide a evidence base regarding the production of biofuels and biochemicals from the sugar platform, offering the following outcomes:

- An assessment of the status of the different pathways, mapping their suitable feedstocks and potential products, and identifying technology opportunities, enablers and barriers to commercialization;
- An assessment of European developments, and how competitive European industry is likely to be versus other world regions;
- For a defined set of ten case studies, an analysis of production costs, and comparison of business cases against current technologies in the market;
- A sustainability assessment using key criteria such as GHG emissions, land use, safety issues, and other environmental and socio‐economic factors;
- The identification of current research gaps and R&D needs—with a focus on recommending measures that will accelerate the introduction of large‐scale demonstration facilities.

This study on the sugar platform selected the 25 most relevant BBPs, out of which the vast majority (16 products) were clearly developed through a biotechnological (biological or intracellular) route. Almost all of these biotechnological BBPs were somehow related to the plastics sector, some being biopolymers (PLA, PHA, bio‐PE) and some others being key monomers for innovative bioplastics (e.g., iso‐butene (which is the building block for elastomers), and 1,4‐butanediol (BDO), which is a building block for biopolyesters, bioelastomers, and biopolyurethanes).

The latest study, named BIOSPRI, focused on a variegated set of biomass components, either available in large volumes (natural rubber, lignins, plant fibers, vegetable oils, and animal fats) or in low volumes but offering high added‐value (terpenes, polyelectrolytes), in addition to urban waste (organic fraction of municipal solid waste and urban waste water) [1]. Similarly to the previous study dedicated to the sugar platform, the BIOSPRI study also clearly evidenced the key role played by biotechnology in the development of innovative BBPs starting from the above‐mentioned selection of biomass platforms. The study offered a mapping of more than one hundred emerging innovations derived thereof, and claimed as a main outcome of the study that bio‐based innovative solutions somehow related to the plastics sector certainly occupy the most relevant positions in the ranking of the top‐emerging ones. In fact, the BIOSPRI study down‐selected the 20 most innovative BBPs out of the overall mapping for the development of detailed case studies. Down‐selection of the top‐emerging BBPs was achieved by applying five assessment criteria to the more than 100 products mapped. Assessment was, respectively, based on (a) level of technological readiness (TRL) at least reaching the pilot scale (i.e., TRL 5); (b) presence of an active marketplace; (c) EU‐based development; (d) degree of innovativeness; and (e) market potential. The final list of the selected top‐emerging 20 BBPs again included many products strictly related to the plastics sector and developed through a biotechnological approach, such as polyamide‐12 (PA12), third‐generation chitosan (also called biotechnological or fungal chitosan), PHA from urban waste, and more.

In the following section, a description is offered of some of the most relevant BBPs described by the BIOSPRI study, which represent significant innovation in the plastics sector and are exclusively derived from biotechnological methods. Bio‐based polyamide 12 (PA12), fungal chitosan and PHA obtained from vegetable oils and animal fats, are three different kinds of biopolymers deserving deployment in the next 5–10 years, and are currently under development at the pre‐industrial scale level thanks to the driving role of biotechnology in the transition from plastics to bioplastics.

## Results from the BIOSPRI tender study on support to R&I policy on bio‐based products

### Polyamide‐12 (PA12)

#### Introduction

Aliphatic polyamides (PA) are industrially synthesized by ring‐opening polymerization of cyclic monomers (lactams) or by step‐growth polycondensation of diacids/diesters with diamines, or ω‐amino acids/esters. PA are engineered polymers, used as high‐performance materials [13], and they include the PA 6 to 12, and PA4,6 and PA6,6, PA6,10, and PA6,12 [14]. As an example, PA6 is synthesized by ring‐opening polymerization of caprolactam and PA6,6 is synthesized by polycondensation of adipic acid with hexamethylenediamine. Some bio‐based PA have been already developed such as PA11, PA10,10, and partially bio‐based variations using the same monomers, commercialized under the trade name Rilsan T® by the company Arkema, or similarly by EVONIK and other suppliers.

Aliphatic polyamides are semi‐crystalline polymers. The regular spatial alignment of amide groups allows a high number of hydrogen bonding to develop when chains are aligned. The crystalline regions contribute to the hardness, yield strength, chemical resistance, creep resistance, and temperature stability, while the amorphous areas contribute to the impact resistance and ductility.

Aliphatic polyamides are versatile plastics for engineering and excellent fiber materials. As per their application, aliphatic polyamides are categorized into two divisions: polyamide fibers and polyamide thermoplastics.

Polyamides fibers are mainly used in carpets, apparel, tire reinforcement, and in other industrial applications. They are used in racing car tires and airplane tires owing to their excellent strength, adhesion to rubber, and fatigue resistance in these demanding applications. Molecular weight, in the case of PA6,6 fibers, is in the range of 12 000–15 000 for apparel fibers and 20 000 for tire yarn is preferred.

Polyamides thermoplastics are important engineering plastics because of their toughness over a wide range of temperatures. In addition, they have good resistance to impact and abrasion, organic solvents, and petroleum products. They are used in many automotive applications such as gears and bearings. Reinforced PA are used for exterior body compartments such as fender extensions, decorative louvers, filler plates, head lamp housings, cross‐over panels, and many other applications. In the electrical and electronic field, polyamides are used in making plugs, sockets, switches, and connectors.

PA12 has a low concentration of amide moieties compared to other commercially available polyamides. PA12 absorbs very little moisture, has excellent resistance to chemicals (e.g., hydraulic fluids, oil, fuels), dampens noise and vibration, and is highly processable.

#### Product description

**Fig. 1:**
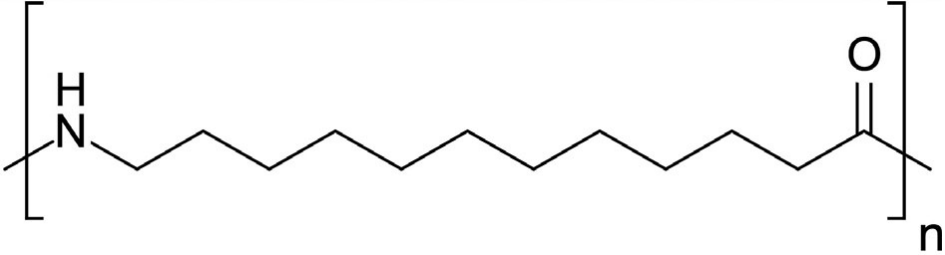
Polyamide 12.

PA12 is a long‐chain linear polyamide belonging to the aliphatic polyamide groups [15] and is characterized by 12 carbon atoms between two nitrogen atoms of the two amide groups (Fig. 1). It presents a melting point of 180 °C and medium to high viscosity grade [16].

The PA12 currently commercialized is exclusively based on fossil sources. It is prepared on an industrial scale via ring‐opening polymerization of lauryl lactam converted after multiple step synthesis starting from butadiene [17]. It can also be prepared from ω‐amino‐dodecanoic acid [18]. UBE in Japan is using 12‐amino‐dodecanoic acid in its manufacturing process for PA12.

For bio‐based PA12, the biomass‐derived monomers ω‐amino lauric acid (ALA) or 12‐amino‐dodecanoic acid (saturated amino acid) are used and they represent the fully renewable alternative to the petroleum‐based lauryl lactam (LL) derived from butadiene. Conventional fossil‐based PA12 and fully bio‐based PA12 offer identical properties apart from the input use and production process; therefore, bio PA12 is to be considered as a drop‐in solution.

At present, fatty acids derived from renewable resources have been proven to be suitable monomers and building blocks for the production of bioplastics, including PA12 [19, 20]. As an example, ω‐amino undecanoic acid, which is synthesized from 10‐undecenoic acid derived from castor oil, is currently used for the industrial production of bio‐based PA11, commercially available from Arkema [17]. The German chemical company Evonik aims to produce bio‐based ALA for the synthesis of PA12; a pilot‐scale plant entered into operation in 2013, in Slovakia. The biotechnological manufacturing process starts from palm kernel oil (PKO) that the company was already using as a platform for the production of other chemicals. PKO contains a large amount of C12 lauric acid and over 50% of it is saturated. The fruit palm tree containing PKO is mainly produced in Asia. Arkema is investigating an alternative process, using the C11 undecenoic acid methyl ester produced from castor oil, and cross‐metathesis with acrylonitrile.

#### Production processes for bio‐based PA12

Three different routes have been proposed for the preparation of fully bio‐based PA12:

- fermentation;
- cross metathesis from C10 or C11 unsaturated esters;
- from bio‐based butadiene, through the synthesis of the monomer dodecanelactame.

The fermentation method is the one completely related to the biotechnological approach, while the second and third methods take advantage of the application of traditional chemical treatments for the synthesis of bio‐based PA12.

As an example, in the bio‐based butadiene route, the major issue is that bio‐butadiene becomes available, but after that there is no need to change the standard production process which is currently applied to petrol‐based butadiene for the synthesis of PA12. Here, butadiene is treated with a Ziegler‐type catalyst system to yield the cyclic trimer, cyclododeca‐1,5,9‐triene. This may then be hydrogenated to give cyclododecane, which is then subjected to direct air oxidation to give a mixture of cyclododecanol and cyclododecanone. Treatment of the mixture with hydroxylamine yields the corresponding oxime, which on treatment with sulfuric acid rearranges to form the monomer dodecanelactame [21].

In the cross‐metathesis approach, the C12 amino‐ester can be produced through olefin metathesis. The successful application of cross metathesis to synthesize PA12 precursors from oleic acid has been reported by the literature. For example, the method reported by Rupilius & Ahmad [22] provides a short and simple route for production of PA12 from PKO. In addition, Abel and coauthors [23] demonstrated that cross metathesis could be applied directly to crude fatty acid methyl ester extracts from algal biomass. However, while metathesis can be regarded as bio‐based process since the raw material used is renewable, the underlying chemical reactions could create more undesired by‐products and hazardous waste than alternative organic reactions.

A completely renovated approach is offered by biotechnology through the fermentation route. The process starts with the vegetable PKO, which generates a new alternative PA12 precursor which is ALA‐equivalent, namely ω‐amino‐dodecanoic acid methyl ester (ADAME). ADAME can be considered as the renewable alternative to petroleum‐based LL. ADAME can be subsequently polymerized to an identical PA12 biopolymer with respect to the one obtained from ALA. This new C12 monomer was obtained from lauric acid methyl ester via *Escherichia coli* bacterial fermentation [15] via a three‐step cascade, in which bio‐based dodecanoic acid methyl ester (DAME) hydroxylation and alcohol oxidation both catalyzed by the alkane monooxygenase AlkBGT from *Pseudomonas putida* GPo1 were followed by terminal amination by means of *Chromobacterium violaceum* ω‐transaminase CV2025. Thereby, dodecanedioic acid monomethyl ester (DDAME) was formed as a major by‐product.

#### Biomass yield and feedstock availability

Palm oil is a plant that produces two different types of oils, the palm oil from mesocarp and PKO from the seed of kernel with different chemical and physical properties. The fatty acid (lauric saturated C12) content in PKO is about 48.2% [24]. One of the advantages compared to other crop oil is that palm oil yields about from 4.09 to 0.5 tonne per ha of PKO [25], which sound competitive compared to the 0.37 tonne per ha of soybeans oil, 0.5 tonne per ha of sunflower oil and 0.75 tonne per ha of rapeseed oil. In terms of availability, nowadays Indonesia and Malaysia account for 85% of global palm oil production. Indonesia is expected to continue to be the leading producers with expansion in land for palm oil; on the other hand, in Malaysia expansion is expected to slow in view of limited land availability. Nevertheless, more and smaller producer countries have emerged within the palm oil market, including Cameroon, Colombia, Costa Rica, Cote d‘Ivoire, Ghana, Guatemala, Honduras, Nigeria, Papua New Guinea, and Thailand.

#### Commercial relevance and future perspectives of bio‐based PA12

Important drivers leading the current and future trends in PA markets are increasing oil prices, that will make conventional polymers more and more expensive, and the expected increase in automotive sales in emerging economies [26]. The PA market at the global level is projected to reach USD 30.76 Billion by 2021, at a CAGR of 4.1% from 2016 to 2021 [27] and USD 32.7 billion by 2025 [28]. In general, the demand for the fastest growing type of sector of the polyamide market by 2021 is projected to be the bio‐based and specialty polyamide sector [27]. While the global PA market was valued at USD 25.14 Billion in 2016 [26], the bio‐based PA market was valued at USD 29 454 million in 2012 and USD 110.5 million in 2016 [27]. The largest market is represented by the Asia‐Pacific area and is followed by Europe and then North America where we find the fastest growing sectors of automobile, packaging, electronics, and consumer goods, including retail. The Chinese, Japanese, Indian, Brazil, and Russian markets are also driving the growth of this market. The growth of the transportation industry due to rising disposable income levels, mainly in China and India, is likely to drive regional markets. This picture is expected to be preserved in the future and will still be dominated by the Asia‐Pacific region, which shows the highest growth rate, although new market alliances will possibly stabilize the overall business. Korea, Taiwan, and Japan are expected to create important opportunities for specialty PA manufacturers, due to increasing demand in PA 12‐based applications. On the other hand, Central and South America will show only moderate growth in the future [26].

#### Value proposition and sustainability

It is ambiguous whether PA based on renewable raw materials are in fact more sustainable than other types when considering factors such as landscape consumption [29], use of agrochemicals and fertilizers, water, transport, working conditions, and the impact on food security [30].

In terms of sustainability, consumers are demanding verification of the way palm oil has been produced. The Round Table of Palm Oil Supply Chain Certification systems addresses this issue by reducing the risk of nonsustainable palm oil use by consumers while further driving the mainstream trade of sustainable palm oil, according to the company [31] (Green Chemical Blog, 2015). In order to allow future sustainable development of the palm oil industry, it is crucial to consider the increment of palm tree yield per hectare and the value addition of the oil. At the same time, it is important to reduce production costs. In this respect, special attention should be given to the improvement of oil palm varieties with higher yields and good oil quality, and which are compact in architecture, better adapted to climate change and exhibit higher tolerance to diseases [32, 33]. Alternative vegetable oils to support the process have been tested, such as coconut oil [34], but their current technology readiness level is still lower with respect to the PKO route. Switching from fossil‐based to bio‐based PA12, sustainably produced, would support the economies of countries growing palm oil, in addition to reducing the carbon footprint of the wide variety of final products that are produced using PA12 as input.

### Third‐generation Chitosan

#### Introduction

Chitin is a very abundant biopolymer in nature [35], being the second most abundant polysaccharide after cellulose. It can be obtained from many sources such as exoskeletons of crustaceans, clams (endo‐skeleton of cephalopods), cell walls of fungi and microalgae, insect exoskeletons, yeast, and the spines of diatoms [36, 37, 38]. At present, crustacean shells represent the most important source of chitin for industrial production, due to the availability of marine waste material from the seafood processing industry [39], and well‐established chemical processes of deacetylation. In fact, chitin use on a large scale is limited due to its water and (most) solvent insolubility. Deacetylation to chitosan represents the most relevant modification of chitin to a water‐soluble derivative, with much wider potential applications. Different degrees of deacetylation are possible, and therefore several grades of chitosan can be derived from chitin, offering a set of different chemical, physical, and mechanical properties [40, 41].

The interest in chitosans as advanced functional biopolymers comes from their special features which make them suitable for specialty applications and development of high added‐value polymer solutions. Biomedical applications are of primary importance because of their biocompatibility, biodegradability, and nontoxicity [42], added to their intrinsic antimicrobial activity [43] and low immunogenicity, which clearly point to an immense potential for future development. These biopolymers can be easily processed into gels, sponges, membranes, beads, and scaffold forms [44]. Chitosan‐based nanomaterials, including nanofibers, nanoparticles, and nanocomposite scaffolds for tissue engineering [45], wound dressing, drug delivery [46] and cancer diagnosis represent huge potential for future development in advanced materials for medical use. Chitosans also find relevant uses in other sectors, such as bioremediation [47, 48] and food preservation [49].

#### Product description

**Fig. 2:**
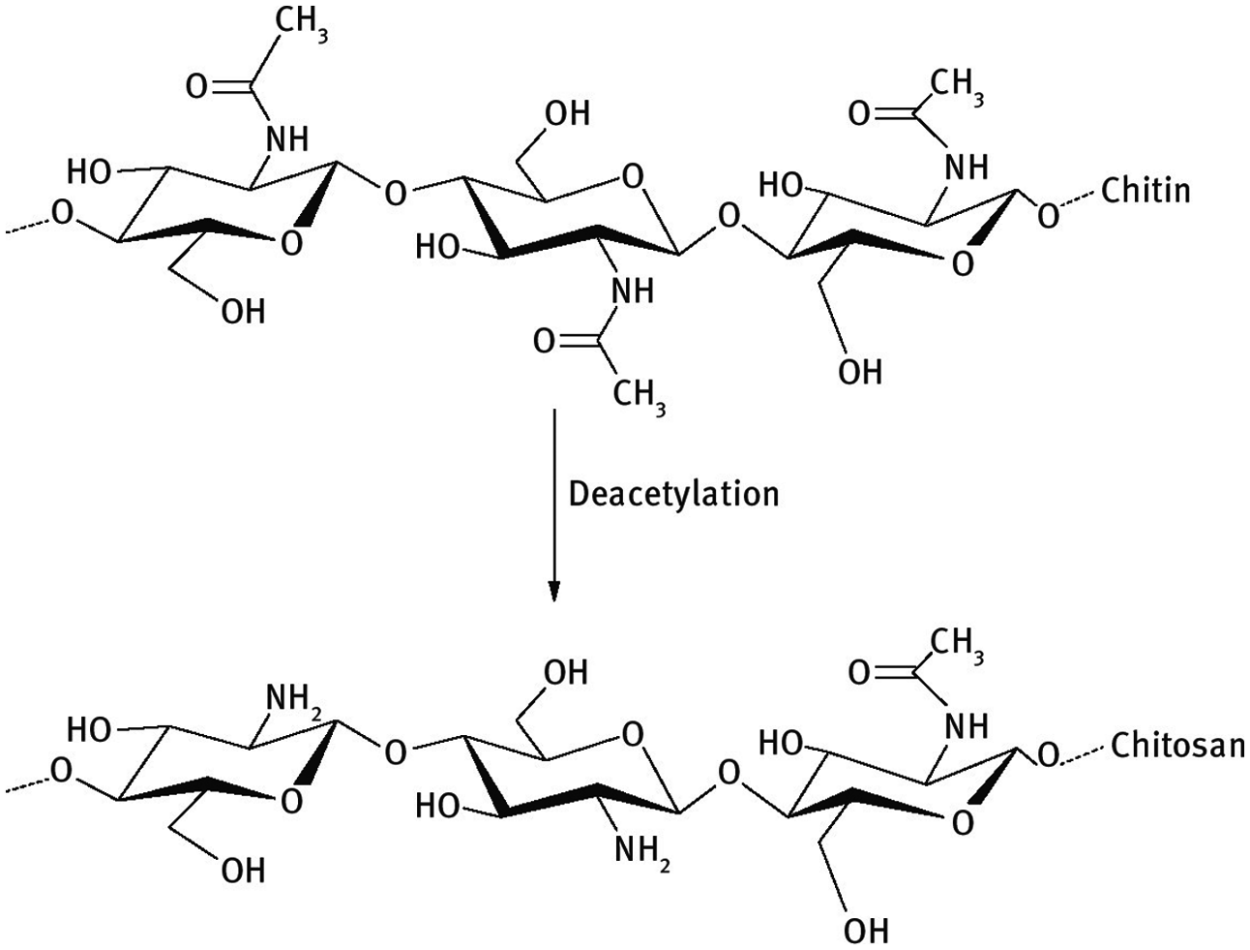
Chitin and chitosan.

Chitosan, classified by the European Commission as a ‘basic substance’ in 2014, is a renewable polysaccharide. Specifically, it is an aminoglucopyran. It is a linear cationic heteropolymer (Fig. 2). In its dissolved form, only achieved at ph < 5,7, thanks to the positive charge distributed along the biopolymer chain, chitosan gives rise to versatile uses based on its chelating, antimicrobial, gelling and film‐forming properties [50].

With reference to the source biomass and the chemical characteristics, three kinds of chitosan can be defined:

- Chitosans derived from animal chitin of marine origin, deacetylated through chemical (soda) treatment is referred to as *first‐generation chitosan*. These chitosans benefit from a low‐cost, easy‐processing route, but suffer from scarce control over the degree of deacetylation and the broad distribution of the biopolymer molecular weights [51].
- If chitosans come from the enzymatic processing of animal chitin, then they are called *second‐generation chitosans* [52]. These biopolymers are better defined than 1st generation chitosan in terms of their degree of polymerization and acetylation, and are therefore more suitable for the development of reliable products with good batch‐to‐batch consistency for industrial applications. A mix of molecules showing different chain length, varied degree of acetylation, and different acetylation patterns characterize both first‐ and second‐generation chitosans.
- Finally, third‐generation chitosan, also called biotechnological or fungal chitosan, is a high‐purity non‐animal‐sourced biopolymer, derived exclusively from renewable, non‐GMO sources such as algae and fungi, without any synthetic manipulations [53]. Source biomass is mainly represented by waste from agro‐alimentary industries. Regarding the chemical characteristics, the molecular weight and degree of deacetylation of fungal chitosan can be controlled by varying the fermentation conditions, while it is rather randomly obtained with chemical treatment of animal‐sourced chitin. Specifically, compared with chitosan from crustaceans, characterized by a high molecular weight (1.5 × 10^6^ Da), fungal chitosan produces a medium‐low molecular weight (1–12 × 10^4^ Da) with molecular weight homogeneity [54], and nonrandom patterns of acetylation, and therefore a well‐defined chemical structure, known cellular modes of action, and better defined biological activities [55]. It is expected that these chitosans will create important opportunities for the market in the future for advanced applications, mainly in the medical field, due to its high purity and a well‐defined chemical composition. Furthermore, as regards raw material supply, due to dis‐continuous supply and seasonal variations of marine sources, the use of fungi could represent an advantageous alternative.

The overall differences between first‐ and second‐generation chitosans compared to fungal chitosan are reported in Table 1.

**Table 1:** Generations and properties of chitosans.

Chitosan generation	Characteristics	Source biomass	Applications
1st/2nd generation	Animal derived (crustaceans) High molecular weight (1.5 × 10^6^ Da) Random patterns of acetylation Low purity Seasonal variation	Marine wastes	Nonadvanced applications (i.e., agriculture, wastewater treatment, and in general all the applications that not require high volume and/or high purity)
3rd generation	Nonanimal derived (derived from fungi and algae) Medium‐low molecular weight (1‐12 × 10^4^ Da) Low molecular weight homogeneity Nonrandom patterns of acetylation Shrimp‐protein and heavy metal free High purity Nonseasonal variation	Wastes from agro‐alimentary industries and from Biotech industries	Advanced applications (i.e., medicine, cosmetics, health care) and when a high and constant quality is strictly required

The properties of fungal chitosans compared with those of a commercial chitosan derived from crab shells are shown in Table 2. The degree of deacetylation is an important parameter affecting the physicochemical properties of chitosan; higher degrees of deacetylation induce higher concentration of positive charges along the polymer chain. This characteristic makes the biopolymer more suitable for food applications as a coagulating or chelating agent, as clarifying agent or as antimicrobial agent. Chitosan with lower molecular weight was reported to reduce the tensile strength and elongation of the chitosan membrane but to increase its permeability. Chitosan from fungi and algae shows more homogeneity in terms of chemical composition but at present is less available and more expensive compared to marine chitosan. To date, small quantities of these molecules are made available on the market; few attempts have been made to obtain chitosan with reliable and predictable biological functionalities in amounts that are appropriate to the market request. It is currently not feasible for high‐volume and low‐cost applications.

**Table 2:** Properties of fungal chitosans [57].

Chitosan from	Degree of deacetylation (%)	Molecular weight (Da)	Viscosity (cP)
Crab shell	97.9 ± 0.9	9.4 × 10^5^	372.7
*Aspergillus niger*	90.0 ± 2.1	1.4 × 10^5^	6.2
*Rhizopus oryzae*	87.9 ± 2.1	6.9 × 10^4^	3.5
*Lentinus edodes*	86.5 ± 2.2	1.9 × 10^5^	5.8
*Pleurotus sajo‐caju*	83.8 ± 0.1	1.1 × 10^5^	5.6
*Zygosaccharomyces rouxii*	85.1 ± 1.1	2.7 × 10^4^	3.3
*Candida albicans*	83.8 ± 0.8	1.1 × 10^5^	3.1

#### Production process

Chitosans can be produced by several species of fungi. The most common method for the production of chitosan from mycelia of fungi involves fungi growth by solid‐state fermentation on carbon‐rich substrates, mainly agro‐waste such as sweet potatoes or others. The chitosan is then extracted by enzymatic treatment [56]. Species of filamentous fungi, such as *Aspergillus niger*, *Rhizopus oryzae*, *Lentinus edodes,* and *Pleurotus sajo‐caju*, but also yeast strains, such as *Zygosaccharomyces rouxii* TISTR5058 and *Candida albicans* TISTR5239, were investigated for their ability to produce chitosan in complex media [57]. A remarkable characteristic of the composition of zygomycetes is the high concentration of chitosan in their cell walls. In *R. oryzae*, it amounts to 42% of the total cell mass [58], and deriving chitosan from these fungi might thus offer an alternative to chitosan production involving deacetylation of chitin from marine crustacean shells. Deacetylation of crustacean shells is normally conducted through harsh alkaline hydrolysis at high concentration and temperature, which entails a long processing time, environmental pollution, and inconsistent physicochemical properties of the produced chitosan. Production of chitosan from zygomycetes under milder controlled conditions could yield a readily available and much more consistent product. Fungal chitosan was produced at 10–140 mg·g^−1^ cell dry weight and had a degree of deacetylation of 84–90% and a molecular weight of 2.7 × 10^4^–1.9 × 10^5^ Da with a viscosity of 3.1–6.2 centipoises (cP). *R. oryzae* TISTR3189 was found to be the producer of the highest amounts of chitosan, suitable for commercial deployment of this production strategy for chitosans.

#### Biomass yield and feedstock availability

As compared to marine sources, chitosan production using fungi is minor, and the fungi showing the highest portion of chitosan appears to be the *mucoraceous fungi* [54, 57].

Chitosan content strongly varies and depends on the specific species of fungi, as reported in Table 3. According to Ghormade *et al*. [54], there exist three major sources of fungi that can be exploited at a commercial level: (a) waste fungal biomass from biotech industries where thousands of tons of waste fungal are produced per year, (b) fungi containing high amounts of chitosan grown by fungal fermentation, and (c) value addition to existing *mycotech* products.

**Table 3:** Chitosan content in different fungi species [57].

Fungal species	Chitosan content (%)
*Aspergillus niger*	11
*Rhizopus oryzae*	38
*Lentinus edodes*	3.3
*Zygosaccharomyces rouxii*	3.6
*Candida albicans*	4.4

In the first group of sources, it can be mentioned that more than 80 000 tons of waste *A. niger* biomass are generated per year from the production of citric acid. Additionally, it can be estimated that in the brewing and baking industries where *S. cerevisiae* and *S. carlbergenis genuses* are traditionally used, biomass availability is more than 130 tonne yeast lees/year. For penicillin production, 1 tonne of penicillin implies around 8–10 tonne/year of waste that can be used for chitosan production.

In the second group of sources involving fermentation of fungus, major contents can be found in *Gongronella butleri*, *Mucor rouxii*, and *Absidia coerulea*, amongst zygomycetous fungi. More specifically, *Benjaminiella poitrasii*, *Cunnighamella blackesleeanus*, *Gongrenella butleri*, *Mortierella isabelina*, *Rhizopus delemar*, and *Rhizopus stolonifera*, amongst others, show a chitosan content ranging between 6.7% and 10.4% production.

#### Commercial relevance and future perspectives

The current amount of third‐generation chitosan produced is currently very low so that market trends are extremely hard to forecast. However, both the chitin and chitosan markets, at the global level, have been growing massively in recent years, due to the expansion of their application domain, which is even larger if co‐products are considered. Applications include biopharmaceutical, cosmetics, biotechnological and biomedical, agriculture, and both food and nonfood industries.

Some features justify positive expectations for the future, such as low toxicity, excellent biocompatibility, versatile biological activity, and complete biodegradability. In addition, the third‐generation chitosan derived from fungi and algae presents many advantages compared with 1st and 2nd generation, primarily less allergenic tendency. Other factors supporting the market development of chitosan are as follows:

- The enhancement of the water treatment sector resulting from high demand for removal of metals and chemicals from wastewater, including pesticides, surfactants, phenol, and polychlorinated biphenyls.
- The demand for the compound in food and beverage application in Europe, which is expected to grow at significant rate due to rising demand for the wine‐processing industry.
- The development expected in the cosmetics industry due to the rising demand for bio‐derived personal care products. The cosmetic industry was the largest application sector of chitosan in North America in 2015 [59].

On the other hand, the growth of value‐added chitosan‐based products is limited by the availability of a sustainable supply chain: the process chemistry for bulk chitosan manufacturing is currently not very environmentally friendly. Green technologies for chitosan modification are facing the challenge of economic viability which hinders its current development. According to existing analysis, we can consider a CAGR of 14% after 2020 to be reasonable, declining over the following years due to a contained effect of market saturation, assuming a $2.0 billion volume in 2016 and 155 000 metric tons of volume reached in 2022 [60].

Price estimates for chitosans and chitin are not clear and notably vary amongst the studies analyzed. However, the source of chitosan derivation strongly affects final price; also, chitosans derived from fungi and algae are notably more expensive than animal‐sourced ones. Chitosan from fungal sources is less available compared to marine chitosan sources, which implies higher production costs and therefore very high sale price. A current share of 0.2% of nonanimal chitin is envisaged for the total chitin market, growing slightly over time as soon as production costs are able to decrease. Overall price dynamics could mainly depend on two opposite forces. On the one hand, the increasing demand for high‐quality chitosan products will put pressure on their prices. On the other hand, expected improvements in technologies and chemical processes will reduce marginal production costs and limit the price increase. A modest annual price increase is therefore expected in time (1%).

#### Value proposition and sustainability

The production of chitosan from fungi can be made more economically advantageous by the exploitation of a varied set of possible co‐products and by‐products. As reported by Kuk [61], both chitin and chitosan can be used as starting materials for the production of high added‐value chemicals, such as mono‐, di‐, and oligosaccharides that are derived with additional hydrolysis processes [62]. Examples are chitobiose, the N‐acetylglucosamine monomer, and glucosamine salts via enzymatically catalyzed hydrolysis reactions of the obtained chitin/chitosan.

An important implementation of chitosanase is the preparation of chitosan oligosaccharide from chitosan [63], which is able to prevent the accumulation of fat in internal organs, plays a remarkable role in liver function and in stimulating the immunological system, and may find pharmaceutical application in formulations for the control of cholesterol accumulation.

For mushroom industry waste, where stalks, which represent ~ 25–33% of the weight of fresh mushrooms, are normally used as low economic value animal feed, a trade‐off exists. Indeed, this biowaste material could be utilized to produce vitamin D and chitosan as co‐products of the industry of high‐quality mushrooms [64].

Therefore, it is clear that one of the most relevant aspects related to the production of chitosan from fungi is that large amounts of waste can be valued, and seasonal availability of marine sources can be overcome through the rapid, easy, and cheap cultivation of fungal species.

Chitosans, recently used in wastewater treatment to remove heavy metals from polluted sediment [65], also respond to the increasing need of addressing environmental and pollution problems relative to rivers and lakes showing sediment contamination. On the same path, chitosans with a high deacetyletic degree can support lower food waste as they show potential for extending the shelf life of refrigerated fish fillets, due to their inhibitory properties, thus contributing to mitigating the global problem of food loss [49].

### PHA from renewable oils and fats

#### Introduction

Polyhydroxyalkanoates (PHA) are intracellular biopolyesters accumulated as a carbon supply or for energy storage by various microorganisms. The homopolymer poly(3‐hydroxybutyrate) (PHB) and its copolymers containing valerate units (PHBV) or hexanoate units (PHBH) represent the most diffused types of PHA. PHA macromolecular chains can be synthesized from numerous carbon‐rich substrates by the biosynthetic action of selected prokaryotic microorganisms. Sugar‐rich substrates, such as sugar beets molasses or sugar cane bagasse, have been widely studied for the industrial production of PHA [12]. All industrially relevant production worldwide is based on these kinds of food sources for microorganisms (http://www.bio‐on.it. http://www.biomer.de. http://www.kaneka.co.jp). The function of PHA granules inside bacterial cells is that of carbon and energy storage, which can be degraded when necessary by several microorganisms producing depolymerizing enzymes. This makes PHA degradable to water and carbon dioxide (or methane, under anaerobic conditions) in all biologically active environments such as soil, open waters (i.e., rivers and lakes, seas and oceans),compost, and sewage [66, 67].

A variety of waste streams different from sugars have been tested in earlier decades to produce PHA in economically sound ways. Examples of used carbon sources are different kinds of agro‐industrial food waste [68, 69, 70], organic municipal waste [71], and activated sludge coming from waste water treatment [72].

It is noteworthy that the molecular structure of PHA chains accumulated inside bacterial cells, and their consequent physical and mechanical properties as plastic materials, does not directly vary based upon the kind of biomass feedstock used as a carbon source for bacterial fermentation. It is only dependent on the bacterial stream used for the fermentation process, its operative conditions, and the number of carbon atoms available in the chemical moieties of the feedstock, which are used as building blocks by bacteria.

In the search for alternative carbon sources for PHA bacterial fermentation, which may be more affordable with respect to sugars, attention has focused on vegetable oils [73, 74] and animal fat waste, such as residues from slaughterhouses [75], and glycerol as the main residue from biodiesel production [76, 77], but also carbon‐rich wastewater from different industrial activities [78].

#### Product description

The chemical and physical properties of PHA biopolymers are very similar to those characterizing petroleum‐derived commodity polymers, such as polypropylene and polyethylene. This makes PHA a very good substitute for conventional plastics [79, 80], and their uses and applications can vary accordingly to their specific molecular structure and formulation with additives.

Depending on the chain length in the PHA subunit (monomer), the hydrophobicity and a number of other properties including the glass transition temperature, the melting point, and level of crystalline color, can vary. Normally, short‐length PHA are hard crystalline materials; medium‐chain length PHA are ductile plastics and have a much lower melting point and glass transition temperature; and PHA with longer pendant groups are rather elastomeric.

Notwithstanding the bacterial strain and carbon‐rich substrates selected for their synthesis, PHA always show spontaneous and complete biodegradability in several environmental conditions, ranging from composting industrial and home facilities to open waters, that is, rivers, seas, oceans, and wet lands. This makes PHA particularly attractive because this feature of complete biodegradability is coupled with their 100% renewable and biotechnological origin [81].

For the homopolymer 3‐hydroxybutyrate) (PHB), which represents the most‐studied example of biodegradable polyesters belonging to the family of PHA, its use is mainly as a substitute for rigid commodity plastics, such as HDPE, PP, and ABS. It is highly crystalline, and therefore, it is optically opaque, highly stiff, and resistant to tensile stress. PHB suffers from being quite brittle, and its processing on standard industrial equipment can be achieved upon optimization of processing parameters, mainly to accommodate its rheological and thermal properties. Several PHB‐based grades, fully or partially biodegradable, can be developed by polymer modification or melt blending with several other commercial plastics and bioplastics. The resulting level of biodegradability depends on the composition of the blend.

At present, PHB still has higher production costs with respect to its fossil‐based commodity plastics (HDPE and PP mainly), but it holds advantages mainly related to the full biodegradability, renewable origin, and possibility to be obtained by the valorization of waste.

The most important features of PHA are its biodegradable capacity under aerobic and anaerobic conditions. Degradation often occurs upon exposure to soil, compost, or marine sediment. However, the biodegradation rate depends on factors such as exposed surface area, moisture, temperature, pH, and molecular weight.

Biocompatibility is perhaps the most important property for applications in the medical field [82, 83]. In addition, biopolyesters are inert, water‐insoluble, not affected by moisture and indefinitely stable in air. In pharmaceutical applications, PHA synthase is the key enzyme for PHA biosynthesis and the same can be exploited for the development of many chiral derivatives [84].

Moreover, the food and beverage industries take advantage of PHA satisfactory barrier properties. Water‐resistant surfaces, moisture vapor barriers, and UV‐resistant layers based on PHA can be used in the packaging industry [85]. Last but not least, PHA may also find relevant applications in engineering sectors, such as sensors [86, 87] or construction [88].

#### Production process

Microbial PHAs are bacterial polyesters produced by enzymatic reactions inside microbial cells from acetyl‐co‐enzymeA (acetyl‐CoAs) by PHA synthase, which is a substrate‐specific enzyme present in the cell cytosol. PHA derived from animal fat and vegetable oils can be synthesized by different microorganisms [89]. Microorganisms able to transform triglycerides into PHA polymer chains, accumulated in the form of granules inside the bacterial cell cytosol, should be characterized by two main features: being able to produce a lipase enzyme to hydrolyze the triglycerides to liberate the long‐chain fatty acids, and to then be transformed into PHA chains by β‐oxidation. In fact, if the carbon substrate used are oils or lipids and are utilized though the fatty acid pathway, then the oxidation of enoyl‐CoA to (R)‐3‐hydroxyacyl‐CoA takes place due to catalyses initiated by the (R)‐specific enoyl‐CoA hydratase (PhaJ). (R)‐3‐hydroxyacyl‐CoA acts as a substrate for the PHA synthase (PhaC) enzyme and is the immediate precursor of PHA biosynthesis [89, 90].

The use of vegetable oils in the production of PHA is rather straightforward, and almost no kind of pretreatments are required [89, 91]. Any remaining food traces will eventually contribute to the overall carbon supply for the microorganisms. This represents a remarkable difference with respect to biodiesel production, in which waste oils need to be pretreated using esterification and filtration before being used.

The most efficient bacterial strains identified for the intracellular accumulation of PHA using oil and fat carbon substrates are *Cupriavidus necator H16, C. necator ATCC 17699, R. eutropha Re2133*, and *C. nectator* *Re2058/pCB113* [89], all of which show an accumulated PHA content higher than ~ 70% with respect to the dry cell weight. Maximum values of rather 90% (w/w) were observed for *C. necator*, which is the top performing strain when vegetable oils are used as carbon substrates to feed the fermentation process [89].

Solid animal fats, characterized by triacylglycerol‐containing long‐chain fatty acyl groups, require triacylglycerol‐utilizing bacteria, which can secrete lipases. Lipases will release fatty acids in the fermentation media, which are then transformed into PHA polymer chains through β‐oxidation [89, 92]. In general, microorganisms find it more difficult to utilize animal fats than liquid vegetable oils, mainly due to the physical solid state of animal fats at room temperature and under fermentation conditions. Thus, pretreatment of animal fats is usually necessary to bring them into a more accessible physical state, that is, liquid; this can be made, for instance, by pre‐emulsification and heating.

In 1996, Cromwick [93] found that *P. resinovorans* could produce PHA polymers on unhydrolyzed tallow, with a PHA content of 15% of the cell dry weight. However, the ester of the fatty acids from tallow with methanol can be fermented using *P. citronellolis*, with a productivity for medium‐chain length *mcl‐*PHA of 0.036–0.050 g/(L*h) and PHA contents of 20.1–26.6% (wt) [94].

This demonstrates that PHA can be also produced from saturated biodiesel fractions (SFAE) stemming from waste animal fats. This waste biomass source is available in notable amounts in Europe and in other parts of the world; in Europe alone, animal lipids from slaughtering and the animal‐processing industry amount to more than 500 000 t per year. Separating the SFAE fractions from biodiesel enhances its performance as a fuel, while the recovered waste fractions can be advantageously valued as feedstock for the biotechnological production of PHA [94].

#### Biomass yield and feedstock availability

There are many potential advantages in using plant oils instead of sugars as carbon sources for PHA production. The superior source biomass yield is of course of primary importance for a possible industrial up‐scaling of the process. While the maximum yield reported for PHA production starting from glucose is ~ 0.3–0.4 g of PHA per gram of sugar, that value increases at 0.6–0.8 g of PHA when plant oils are used for feeding the fermentation process. This is due to the higher content of carbon present in oils per weight compared to sugars [95]. When comparing the efficacy of WCO with fresh vegetable oil as carbon sources, similar values are found for the growth rate of microorganisms and PHA accumulation. Yields can vary from 0.94 g_PHA_/g_oil_ when WCO is used, to the slightly lower value of 0.87 g_PHA_/g_oil_ for fresh vegetable oils. In any case, yields are very high and PHA accumulation stays in the range 85–75% w/w of the dry biomass content, respectively. Therefore, the WCO is slightly more efficient in terms of yield when compared to pure vegetable oils [89, 96].

At the global level, the feedstock availability for production of PHA from oily biomass is extremely relevant. It was estimated that a 10 million gallon per year biodiesel plant would have the potential of producing 20.9 ton PHB [77]. Considerable amounts of oily waste rich in lipid composition are generated from various developed industrial processes, including animal fats, oil mills, dairy foods, and processing of food material. The Energy Information Administration (EIA) estimates that ~ 11 billion liters of waste vegetable oils are generated annually in the USA. In the European Union, the aggregate amount of waste vegetable oils produced per year is accounted as ~ 1 billion liters. In 2010, the United States produced around 2.7 billion tons of tallow and grease. Thus, large amounts of animal fats with a high fatty acid content are being generated and can be used as a potential feedstock for producing PHA [89].

#### Commercial relevance and future perspectives

Amongst the innovative plastic materials currently under development, PHA are considered as the most promising bioplastics, deserving deployment, and increased utilization over the next few years [97].

The PHA market is expected to reach an estimated volume of 45.49 tons by 2027, based on a growth rate of 7.60% for the period between 2020 and 2027 [98]. Increased use of PHA in various application sectors, including high added‐value sectors such as the biomedical and the cosmetics industries, is expected to drive the PHA market in the next years.

Commercial PHA are sold as commodity bioplastics for the large volume production of disposable goods (packaging, and food service items such as plastic straws, cutlery, trays, and bottles) and for agriculture (mulching films, nutrients carriers), and for high added‐value markets such as the biomedical and the cosmetics industry, 3D printing, and chemical additives.

The vast availability of renewable and cost‐effective raw materials such as bagasse, zein, casein, plant starch, and many more is encouraging the growth of the PHA market. Government regulations and policies against single use plastic is a major factor for the growth of the PHA market. Moreover, with the emergence of new raw materials and growth in the Asia‐Pacific region will create further opportunities for the PHA market. The spontaneous biodegradability of PHA under environmental conditions also represents a main driver for the market growth of PHA.

#### Value proposition and sustainability

PHA produced by bacterial fermentation using vegetable oils or animal fat waste biomass perfectly adhere to the principles of CE and circular bioeconomy. In addition to contributing to the reduced depletion of fossil resources, PHA are fully derived from renewable resources, can be produced from different types of biowaste through low impact biotechnological routes, and offer spontaneous biodegradation in an open environment. On the social side, all of these features together induce positive thinking about plastics in modern society, supporting acceptance and understanding of the opportunities by the end‐users. With regard to evaluation of environmental impact, life cycle assessment methodologies have been widely applied to study PHA. The emission of GHGs during production of PHA showed a reduction of up to 200% and use of fossil energy was reduced by 95%, contributing to a reduction in the amount of waste to be managed [89, 99].

## Conclusions

BIOSPRI, a recent tender study on support to R&I policy in the field of BBPs, commissioned by the European Commission DG RTD, selected the 20 most promising BBPs currently under development, and in the framework of this selection, many products are directly related to the plastics sector. The findings are evidence for the strategic role of biotechnology in driving and boosting the transition from fossil‐based plastics to bioplastics obtained from renewable resources. Three relevant examples were discussed in this review: (a) PA12, which represents an innovative bioplastic mainly for engineering applications; (b) fungal chitosan, which offers relevant features for advanced applications in the biomedical field and for decontamination of waters and soils; and (c) PHA derived from vegetable oils and animal fats, which represents an example of bioplastic fulfilling the complete set of requirements of the circular bioeconomy. Biotechnology is the key enabling technology for the development of these innovative biomaterials, and clearly supports their transition to full technological maturity and commercial accessibility. Valorization of waste and forward‐looking management of critical raw materials are main drivers for the further development of the bioplastics here discussed, and biotechnological methods offer increasing opportunities for the whole plastic sector.

## Conflict of interest

The authors declare no conflict of interest.

## Author contributions

PF, FF, LB, FC and DV contributed to conception, design and development of the study. PF, FF, LB, FC, DV, MDE, and DM equally contributed to the organization and writing of the manuscript, revised, read, and approved the submitted version.

### Data accessibility

Data and information used to elaborate this review paper are accessible through the Publication Office of the European Union, at the following link: https://op.europa.eu/en/publication‐detail/‐/publication/15135e98‐81c2‐11e9‐9f05‐01aa75ed71a1/language‐en/format‐PDF/source‐search.
